# Microsurgery for root coverage: A systematic review

**DOI:** 10.12669/pjms.315.7782

**Published:** 2015

**Authors:** Jian Kang, Shu Meng, Chunjie Li, Zhenhua Luo, Shujuan Guo, Yafei Wu

**Affiliations:** 1 Jian Kang, DDS, State Key Laboratory of Oral Diseases, Dept. of Periodontics, Dept. of Periodontics, Tianjin Stomatological Hospital, Tianjin, China. West China Hospital of Stomatology, Sichuan University, Chengdu, Sichuan, China; 2 Shu Meng, DDS, State Key Laboratory of Oral Diseases, Dept. of Periodontics, West China Hospital of Stomatology, Sichuan University, Chengdu, Sichuan, China; 3 Chunjie Li, DDS, Department of Evidence-Based Dentistry, West China Hospital of Stomatology, Sichuan University, Chengdu, Sichuan, China; 4 Zhenhua Luo, DDS, State Key Laboratory of Oral Diseases, Dept. of Periodontics, West China Hospital of Stomatology, Sichuan University, Chengdu, Sichuan, China; 5 Shujuan Guo, DDS, State Key Laboratory of Oral Diseases, Dept. of Periodontics, West China Hospital of Stomatology, Sichuan University, Chengdu, Sichuan, China; 6 Yafei Wu, DDS, State Key Laboratory of Oral Diseases, Dept. of Periodontics, West China Hospital of Stomatology, Sichuan University, Chengdu, Sichuan, China

**Keywords:** Gingival Recession, Microscopy, Surgery, Surgical Flaps

## Abstract

**Objective:**

To evaluate whether microsurgery gains better result in root coverage compared to conventional surgical techniques.

**Methods:**

A number of databases were searched to identify eligible studies from January 1992 to January 2015.

**The following outcomes were evaluated:**

number of sites exhibiting complete root coverage and patients’ esthetic satisfaction.

**Results:**

Four Randomized Clinical Trials (RCTs) fulfilled the inclusion criteria. A pooled estimate from the two RCTs regarding sub-epithelial connective tissue grafts (SCTG) showed significant achievement in complete root coverage in the microsurgical group [relative risk (RR):1.63; 95% confidence interval (CI): 1.12 to 2.36; *P*=0.01] with acceptable heterogeneity. The other two studies were about coronal advanced flap (CAF) with enamel matrix derivative or free rotated papilla autograft and did not qualify for meta-analysis. Patients’ esthetic satisfaction was analyzed only by one study.

**Conclusions:**

Using microsurgical technique for treating gingival recessions may be effective in achieving complete root coverage for SCTG.

## INTRODUCTION

Gingival recession, is defined as apical shift of gingival margin towards cementoenamel junction and consequently results in tooth root surface exposure. Gingival recession is usually associated with anatomic factors, inflammatory conditions, and trauma, and is one of the most common esthetic complaints of patients in periodontal clinics. Esthetic improvement, dentinal hypersensitivity, root caries are all indications for surgical treatment.^1^

A variety of surgical techniques have been used for root coverage; such as sub-epithelial connective tissue grafts (SCTG), guided tissue regeneration and coronal advanced flap (CAF).^1^ The microsurgical technique involves microscope and fine instruments that allow high-level of accuracy. Microsurgery was introduced in the specialty of periodontics in 1992.^2^ Clinical and histological evidence showed that microsurgical technique may result in primary wound closure,^3^-^5^ which could lead to better esthetic appearance and less postoperative discomfort. Though the microsurgical technique have some benefits, not all randomized clinical trials (RCTs) comparing microsurgery with conventional surgery showed clinical efficacy of the microsurgery. A systematic review was carried out to provide the best level of evidence and summarize the existing studies.

## METHODS

### Inclusion and Exclusion criteria

### Types of studies

Only RCTs with a follow-up of ≥6 months were included (including split-mouth studies randomized by quadrants). However, trials that randomized individual teeth or alternate teeth, with the initial selection of the teeth sequence being randomized, were not included.

### Types of participants

Healthy adult patients with a localized gingival recession (no limitation on Miller classifications^6^) who had undergone surgical treatment.

### Types of interventions and comparison

Plastic surgery for root coverage of gingival recession defects comparing microsurgical procedure (using operational microscope and microsurgical instruments) with conventional procedure (without using microscope).

### Types of outcome measures

Primary outcomes: The number of sites exhibiting complete root coverage. Secondary outcomes: Patients’ esthetic evaluation.

### Literature Search

### Electronic search

MEDLINE (via PubMed), EMBASE (via OVID), CENTRAL (for the Cochrane Central Register of Controlled Trials), Cochrane Oral Health Group’s Specialized Register database, and CNKI in China were searched for identifying RCTs, to be included or considered for this systematic review. For dissertations and grey literature, ProQuest and OpenGrey database were searched. Google Scholar was another source of search. Articles from January 1992 to January 2015 without limitation to any language were searched. Following key words were used:

1. Gingival recession
2. (recession NEAR gingiva*) or (recession NEAR defect*)
3. (exposure NEAR root*) or (exposed NEAR root*)
4. 1 or 2 or 3
5. Microsurgery
6. Microscop*
7. Minimal* NEAR invasive
8. 5 or 6 or 7
9. 4 and 8

### Manual search

Journal of Periodontology, Journal of Periodontal Research, Journal of Clinical Periodontology, and International Journal of Periodontics & Restorative Dentistry were manually searched for articles published through January 1992 to January 2015. References of the eligible studies were further browsed for identification of relevant articles.

### Study selection and data extraction

The title and abstract of each article were screened by two of the authors (JK and SM). Full texts of relevant articles were obtained and then assessed for inclusion also by the same two authors (JK and SM). Disagreements were resolved by discussion with a third author (SJG). Finally, studies fulfilling the inclusion criteria underwent data extraction by two authors (JK and SM) independently using data-extraction forms. Unpublished data and details about the trials were obtained by contacting the concerned researchers.

### Risk of bias assessment

Two authors (JK and SM) assessed the risk of bias in included studies. Random sequence generation, allocation concealment, blinding of participants and personnel, blinding of outcome assessment, incomplete outcome data, selective reporting, and other biases were considered.^7^ In case of disagreement, the dispute was resolved through a discussion with a third author (SJG).

### Data synthesis

For continuous data, mean differences (MDs), standard errors (SEs), and 95% confidence intervals (CIs) were calculated for all studies to combine data from parallel and split-mouth studies.^8^

For dichotomous data, pooled relative risks (RRs) and associated 95% CIs were used. Number needed to treat (NNT) was calculated for sites exhibiting complete root coverage when the pooled estimate was statistical significant (P<0.05). Heterogeneity between studies was analyzed through heterogeneity test and I^2^ statistic. *P*>0.1 and I^2^<50% were taken as acceptable heterogeneity, in which case, a fixed-effects model was employed; otherwise a random-effects model was adopted. Meta-analysis was conducted by Review Manager (RevMan) software (Version 5.1).

## RESULTS

**Fig.1:**
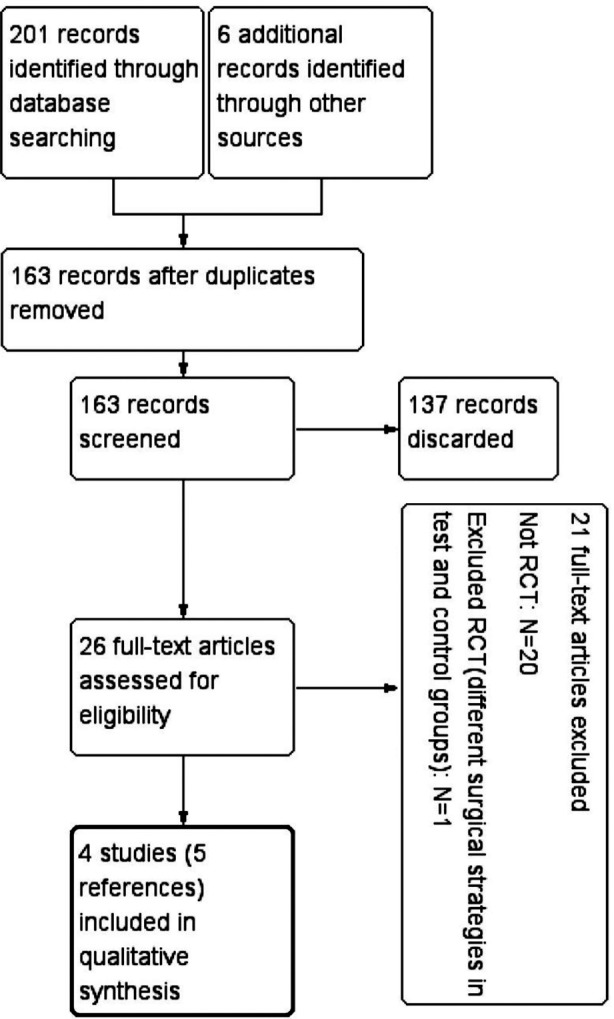
Flow chart of articles screened through the review process.

The results of the search from databases provided 201 records. Six additional records including one dissertation were found by screening the references and Google Scholar. After eliminating the duplicate records, 163 records remained. By screening the titles and abstracts, 137 irrelevant articles were discarded, leaving 26 articles for further evaluation. Full texts of these 26 articles were obtained; of which 20 articles were excluded for not being RCTs.^9^-^29^ One RCT was excluded because the surgical strategies were different in the microsurgical and control groups.^5^ The dissertation by Pandey Suraj Devendra^30^ and the article by Pandey and Mehta^31^ described the same study. The dissertation and article were merged into one.^31^ Finally, four RCTs in five records were included in this review.^3^,^4^,^31^,^32^ A flow chart summarizing the results of the search and reasons for exclusion are listed in Fig.1.

### Characteristics of included studies

Burkhardt and Lang and Bittencourt et al. performed SCTG combined with double papilla flap or a repositioned flap,^3^,^4^ while Andrade performed CAF with enamel matrix derivative.^32^ Pandey and Mehta performed CAF combined with free rotated papilla autograft.^31^ The characteristics of the included studies are summarized as Table-I.

**Table-I:** Characteristics of RCTs.

Study ID	Study design	Participants (n)	Miller class	Interventions	Outcomes	Follow-up (month)
Burkhardt and Lang, 2005	RCT; split-mouth study	10 (Two were lost to follow-up)	I or II	***Test group:*** SCTG+double-pedicle papilla flap under 5× and 15× magnification	1. Postsurgical vascularization; 2. PD; 3. GR; 4. CAL; 5. GI and PI	12
				***Control group:*** SCTG+double-pedicle papilla flap by conventional approach		
Anderade et al. 2010	RCT; parallel study	30	I or III	***Text group:*** CAF+EMD by microsurgical approach	1. GR; 2. PD; 3. CAL; 4. WKT;5. TKT 6. Pain evaluation	6
				***Control group:*** CAF+EMD by conventional approach		
Bittencourt et al. 2012	RCT; split-mouth study	24	I or II	***Test group:*** SCTG with a microscope under 8× to 12× magnification	1.GR; 2. WKT; 3. RW; 4. PD; 5. CAL; 6. TKT; 7. Postoperative pain; 8. Patients’ satisfaction (relative to esthetics, root sensitivity before and after surgery)	12
				***Control group:*** SCTG performed without a microscope but employing fine instruments		
Pandey and Mehta, 2013	RCT; split-mouth study	10	I or II	***Text group:*** CAF+free rotated papilla autograft under 10× magnification	1.Plaque index; 2.Gingival index; 3.GR; 4.RW; 5.CAL; 6.WKT	6
				***Control group:*** CAF+free rotated papilla autograft by conventional approach		

CRC: complete root coverage; CAF: coronally advanced flaps; CAL: clinical attachment level; EMD: enamel matrix derivative; GI: gingival index; GR: gingival recession; PD: probing depth; PI: plaque index; RCT: randomized controlled trial; RW: recession width; SCTG: subepithelial connective tissue graft; TKT: thickness of keratinized tissue; WKT: width of keratinized tissue; NR: not reported.

### Risk of bias in included studies

**Fig.2:**
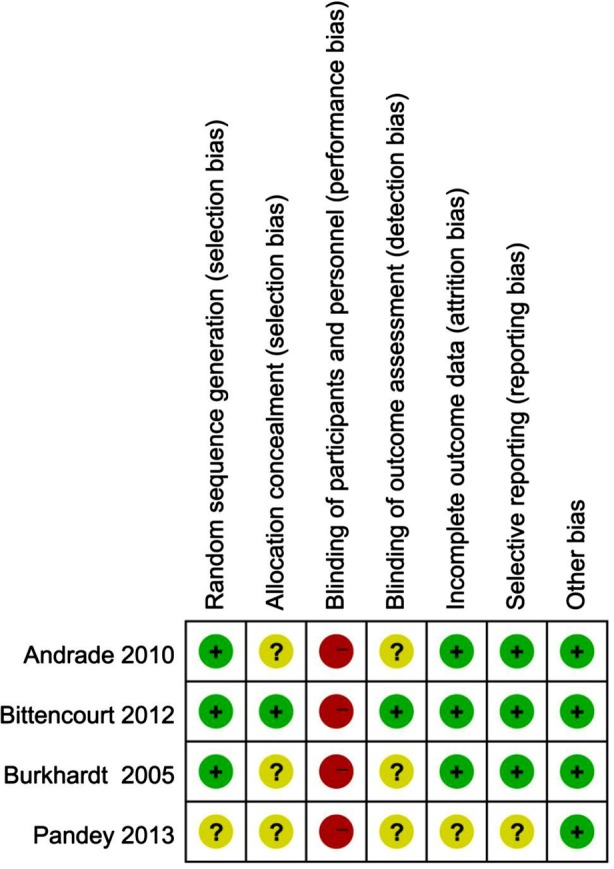
Risk of bias summary: review authors’ judgments about each risk of bias item for each included study.

Risk of bias in the included studies is summarized in Fig.2. All the included trials were categorized as high risk of bias due to the difficulties in blinding patients and surgeons while using operational microscope. Concealment of random sequence was poorly reported by most of the studies.^4^,^31^,^32^ Bittencourt et al were contacted; they responded and explained that they adequately concealed the random allocation scheme.^3^ Lack of details about random sequence generation^31^ and masking of outcome assessors^4^,^31^,^32^ were the other sources of bias. In one study, two patients out of ten could not be followed-up because of relocation.^4^ There might not be great effect on the risk of attrition bias for split-mouth studies due to the balanced dropout numbers with the same reason across groups.^33^ Data for these two patients in the study^4^ were excluded from the analysis. The trial by Pandey and Mehta^31^ had unclear risk of reporting bias for not reporting the sites of complete root coverage which is considered to be an important outcome.

### Effects of interventions

### Number of sites exhibiting complete root coverage

**Fig.3:**
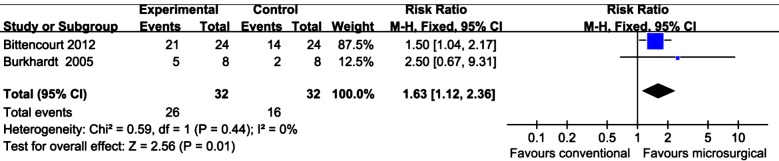
Meta-analysis for complete root coverage by SCTG comparing microsurgery and conventional surgery.

Two split-mouth studies^3^,^4^ employing SCGT and one parallel study^32^ combining CAF with enamel matrix derivative reported the results of complete root coverage. For SCTG, a pooled estimate showed significant achievement in complete root coverage in the microsurgery group (RR: 1.63; 95% CI: 1.12 to 2.36; *P*=0.01) with acceptable heterogeneity (Fig.3). Additionally, the number needed to treat was 4 (95%CI: 1.88 to 10.78), implying that one additional person will gain complete root coverage for every four participants receiving the microsurgical intervention rather than conventional approach. For CAF with enamel matrix derivative microsurgical group achieved 73.3% complete root coverage versus 46.7% in control group (P=0.26).

### Patients’ esthetic satisfaction

Patients’ satisfaction about esthetic improvement was reported only by one study^3^ that was collected through a questionnaire. All patients in microsurgery group were satisfied from the gingival appearance, comparing with 79.1% in the conventional surgery group.

## DISCUSSION

This systematic review evaluated the effectiveness of root coverage operation through microsurgical technique. Four trials met the proposed inclusion criteria, providing data from patients treated with SCTG, CAF combined with enamel matrix derivative, or free rotated papilla autograft.

Complete root coverage is the ultimate goal of gingival recession treatment. By achieving this goal, periodontist can not only get the greatest degree of improvement in appearance, but also reduce the extent of dentinal hypersensitivity.^34^,^35^ The available evidence, stemming from 2 RCTs^3^,^4^ indicate that complete root coverage can be more predictably achieved by SCTG performed with a microscope. This can be explained by accurately mapped incisions, finely elevated flaps, and precisely closed wound without tension leading to primary wound healing.^22^ Nevertheless, very limited data about complete root coverage are available in the literature for other root coverage techniques such as CAF with enamel matrix derivative or free rotated papilla autograft and guided tissue regeneration. The scarcity of data makes meta-analyses difficult and prevent making conclusive evidence-based statements.

Despite the fact that esthetics is considered as a main reason for opting microsurgery,^3^ few studies evaluated the patients’ satisfaction. Even with complete root coverage; the incision scar, gingival color, and gingival margin contour also affect the esthetic appearance. It is the patients who should primarily evaluate the esthetic success of root coverage. There is a need to investigate patients’ own evaluation on esthetic condition of microsurgical technique in future research.

All the trials included in this study were categorized as high risk of bias. The lack of details about randomization, allocation concealment and blinding, can act as sources of bias and affect the accuracy of the results. Knowledge of the intervention by participants may also impact on patients’ esthetic evaluation. Although it is impossible to mask the surgeons; participants’ blinding could be improved by blindfolding during operation.

This systematic review has the following limitations with regard to the robustness of the results: the paucity of RCTs, small sample size, and methodological flaws within the included studies. Microsurgery might be a promising technique in gingival recession treatment. However, further research is required in this area.

## CONCLUSION

The application of magnification and finer instruments in the treatment of gingival recession may predict greater opportunities of achieving complete root coverage for SCTG.
